# Plasticity in the antipredator behavior of the orange-footed sea cucumber under shifting hydrodynamic forces

**DOI:** 10.1093/cz/zoy100

**Published:** 2019-01-11

**Authors:** Nicholas A W Brown, David R Wilson, Patrick Gagnon

**Affiliations:** 1 Department of Ocean Sciences, Memorial University of Newfoundland, St. John’s, Newfoundland, Canada; 2 Department of Psychology, Memorial University of Newfoundland, St. John’s, Newfoundland, Canada

**Keywords:** behavioral plasticity, benthic invertebrate, predator–prey interaction, reciprocal transplant experiment, *Solaster endeca*, wave environment

## Abstract

Marine invertebrates that move too slowly to evade unfavorable environmental change may instead exhibit phenotypic plasticity, allowing them to adjust to varying conditions. The orange-footed sea cucumber *Cucumaria frondosa* is a slow-moving suspension feeder that is preyed on by the purple sunstar *Solaster endeca*. The sea cucumber’s antipredator behavior involves changing shape and detaching from the substratum, which might increase its probability of being displaced by water motion into an unsuitable environment. We hypothesized that sea cucumbers’ antipredator responses would be diminished under stronger hydrodynamic forces, and that behavioral strategies would be flexible so that individuals could adjust to frequent changes in water flows. In a natural orange-footed sea cucumber habitat, individuals lived along a pronounced hydrodynamic gradient, allowing us to measure antipredator behavior under different water flow strengths. We placed purple sunstars in physical contact with sea cucumbers living at various points along the gradient to elicit antipredator responses. We then repeated this procedure in a laboratory mesocosm that generated weak and strong hydrodynamic forces similar to those observed at the field site. Subjects in the mesocosm experiment were tested in both wave conditions to determine if their antipredator behavior would change in response to sudden environmental change, as would be experienced under deteriorating sea conditions. Antipredator responses did not covary with hydrodynamic forces in the field. However, antipredator responses in the mesocosm experiment increased when individuals were transplanted from strong to weak forces and decreased when transplanted from weak to strong forces. Overall, our results indicate environmentally induced plasticity in the antipredator behavior of the orange-footed sea cucumber.

## Introduction

Slow-moving and sessile marine invertebrates that inhabit dynamic environments cannot easily relocate to a more favorable habitat when environmental conditions deteriorate. Instead, individuals may mitigate deleterious effects *in situ* through alterations in morphological, physiological, or behavioral traits that are induced by environmental change, a phenomenon known as phenotypic plasticity (Stearns 1989; Dukas 1998; Price et al. 2003; Padilla and Savedo 2013). For example, when exposed to relatively high wave forces, mutualistic sponges (*Haliclona caerulea*) and red algae (*Jania adherens*) develop larger attachment surfaces and increase organic density, which allows them to better withstand the heightened mechanical stress (Carballo et al. 2006). Similarly, mussels *Mytilus edulis* increase tenacity by producing more byssal threads during the winter months when flows tend to be stronger (Carrington 2002). Even some free-moving marine organisms exhibit phenotypic plasticity when they cannot escape deteriorating conditions. Purple ochre stars *Pisaster ochraceus*, for example, alter their morphology to reduce drag when exposed to higher hydrodynamic forces (Hayne and Palmer 2013). Such adaptations allow slow-moving organisms to withstand environmental fluctuations that more mobile species often avoid (Gibson 2003).

Along with a dynamic environment, slow-moving and sessile marine organisms must also cope with mobile predators. Consequently, a suite of specialized antipredator responses, known as inducible defences, have evolved (Adler and Harvell 1990). When the predation threat is not immediate, prey organisms may produce morphological defences. For example, *Membranipora membranacea*, a colonial bryozoan, produces new zooids with defensive spines soon after exposure to chemical cues from predators (Harvell 1984, 1986). Effluent water from predatory crabs similarly induces shell-thickening in mussels (e.g., *M.* *edulis*, Leonard et al. 1999; Freeman and Byers 2006; *Semimytilus algosus*, Caro and Castilla 2004). Although morphological change can occur relatively quickly (e.g., a cryptic response; Hultgren and Mittelstaedt 2015), it may not occur quickly enough when an attack is imminent. In these situations, behavioral responses may be faster and more successful (Padilla and Adolph 1996; Heynen et al. 2017). For example, scallops contacted by predators produce defensive responses ranging from valve closure to a dramatic “swimming” escape, depending on the perceived level of threat (e.g., *Pecten maximus*, Thomas and Gruffydd 1971; *Argopecten irradians*, Winter and Hamilton 1985); numerous marine invertebrates exhibit similar mobile escape behaviors (Feder 1972).

Defensive behaviors may facilitate escape but are often subject to environmentally mediated trade-offs. For example, freshwater clams *Corbicula fluminea* evade small predators by retracting vulnerable tissues and closing their valves (Saloom and Duncan 2005). While protected, the clams cannot properly ventilate, and, when dissolved oxygen levels become too low, the clams are forced to reopen their valves; thus, clams face a greater trade-off between predation risk and physiological demands when living in hypoxic conditions (Saloom and Duncan 2005). Conceivably, most slow-moving and sessile marine invertebrates in shallow habitats must respond to predators under various environmental conditions, since conditions immediately surrounding the animal are often changing. The aim of the present study was to use complementary field and mesocosm experiments to investigate environmentally induced plasticity in the antipredator behavior of a slow-moving marine invertebrate with a non-centralized nervous system.

Our focal animal, the orange-footed sea cucumber, is a benthic suspension feeder (Klugh 1923). In eastern Canada, it is hunted primarily by the purple sunstar *Solaster endeca*, which removes up to 2% of its population annually (Himmelman and Dutil 1991; Legault and Himmelman 1993; So et al. 2010). Upon attack by a purple sunstar, orange-footed sea cucumbers exhibit a rapid (ca. 2 min) antipredator response: body elongation and contraction, followed, if necessary, by an increase in buoyancy and detachment from the seabed (Legault and Himmelman 1993; So 2009; Gianasi et al. 2015). Some evidence suggests that this response is adaptive. So (2009) noted that, out of 22 attacks by juvenile purple sunstars on juvenile orange-footed sea cucumbers in a laboratory experiment, all 5 sea cucumbers that detached successfully escaped; in contrast, 13 out of 17 sea cucumbers that remained attached were consumed. Additionally, orange-footed sea cucumbers are more likely to exhibit antipredator responses when attacked by purple sunstars than when attacked by other sea stars that rarely or never consume the sea cucumbers (Legault and Himmelman 1993). This tendency to scale antipredator behavior to the level of predation threat further suggests that the response is adaptive (Sih 1986; Helfman 1989; Lima and Dill 1990; Hölker and Stief 2005; Edelaar and Wright 2006). Body shape change and detachment as antipredator responses have also been documented in other holothurians (Francour 1997). Detachment resulting in successful escape from predatory gastropods (*Tonna* spp.) has been reported in *Holothuria scabra* and *Stichopus horrens* (Kropp 1982; Morton 1991), and *Parastichopus californicus* escapes from purple sunstars and sunflower sea stars (*Pycnopodia helianthoides*) by swimming (Mauzey et al. 1968; Shivji et al. 1983). Thus, detaching likely affords orange-footed sea cucumbers the highest chance of escaping a predatory attack.

The risk associated with detaching may vary with a sea cucumber’s hydrodynamic environment. Small sea cucumbers typically live on shallow seabed (<10 m; Hamel and Mercier 1996), possibly because the warmer water at shallow depths provides refuge from sunstars (Legault and Himmelman 1993; So et al. 2010). Indeed, purple sunstars mainly target small (<15 cm in body length) sea cucumbers and generally avoid shallow seabed (<20 m), where water temperatures often exceed the sunstar’s optimal foraging temperature of 6°C (Ursin 1960; Franz et al. 1981; Himmelman and Dutil 1991; Hamel and Mercier 1996; So et al. 2010). However, shallow benthic habitats are also prone to sudden, relatively large shifts in hydrodynamic forces (Denny 1988). On windy days, water turbulence induced by higher energy surface waves can extend deep into the subtidal zone and well into the sea cucumber’s upper distributional range (Harmelin-Vivien 1994; So et al. 2010), where it can cause detachment, injuries, or death in firmly attached benthic animals (e.g., López et al. 2008; Babarro and Carrington 2013). Without the ability to control their displacement while floating in the water column (So et al. 2010), orange-footed sea cucumbers may therefore face a risk of stranding when they detach in response to a predator, or for any other reason, in shallow coastal waters.

Behavioral plasticity might allow sea cucumbers to adjust to changes in the local flow environment. Orange-footed sea cucumbers actively seek moderate flows and move away from strong currents that hinder feeding, expose them to increased drag, and increase their likelihood of dislodgement (Sun et al. 2018). Other holothurians and benthic invertebrates also exhibit behavioral plasticity that reduces their probability of detaching in turbulent water. The sea cucumbers *Thyone aurea*, *Pentacta doliolum*, and *Pseudocnella insolens* respond to increasing hydrodynamic forces by clumping together, which is thought to decrease drag and provide greater surface area for attachment (Barkai 1991). Similarly, under stronger water flows, the sea urchins *Paracentrotus lividus* and *Strongylocentrotus droebachiensis* engage more tube feet to enhance tenacity, or form tighter intraspecific aggregations (Frey and Gagnon 2016; Cohen-Rengifo et al. 2017). Reducing the strength and frequency of antipredator responses might therefore be another strategy that sea cucumbers use to mitigate the risk of being washed away in turbulent waters.

We hypothesized that hydrodynamic forces influence antipredator responses (change in body shape and detachment from substratum) in orange-footed sea cucumbers exposed to purple sunstars. Specifically, we predicted that sea cucumbers would: (1) exhibit weaker or fewer antipredator responses under relatively strong versus weak hydrodynamic forces and (2) increase their responses when moved from relatively strong into weak hydrodynamic forces and diminish their responses when experiencing the opposite. We first conducted a field experiment to test whether the strength of hydrodynamic forces covaried with depth in a natural orange-footed sea cucumber habitat, and whether antipredator responses of sea cucumbers covaried with the strength of hydrodynamic forces along the gradient. We then brought sea cucumbers from our field site into a laboratory mesocosm to isolate possible effects of hydrodynamic forces on antipredator responses. Midway through the mescosm experiment, we conducted a reciprocal transplant experiment to test if individuals respond to relatively sudden shifts in hydrodynamic forces similar to those observed under rapidly deteriorating sea conditions.

## Materials and Methods

### Study site and subjects

We tested antipredator responses of orange-footed sea cucumbers at, or collected from, Bread and Cheese Cove (47°18′30.8″ N, 52°47′19.1″ W) on the north shore of Bay Bulls, Newfoundland, Canada. The bedrock seabed at Bread and Cheese Cove is colonized by grazing-resistant, red coralline seaweeds (mainly *Lithothamnion glaciale*). The green sea urchin, *S. droebachiensis*, maintains the biological communities in a “barrens” state (*sensu*Lawrence 1975) for most of the year (Blain and Gagnon 2014; Frey and Gagnon 2015). We monitored sea cucumbers naturally distributed along a 110-m-long depth gradient ranging from the turbulent shallow environment at a rocky point on the north shore of the cove to the calmer deeper waters located near the middle of the cove to the south.

Subjects in the field experiment were 16 orange-footed sea cucumbers of similar sizes (see below) selected haphazardly at depths of 5–11 m. We marked each subject by placing a unique numbered lead weight within 30 cm of its location, and by removing neighboring sea cucumbers from within a 1-m radius. One subject (depth: 10.6 m) was excluded from the final sample because it detached from the seabed following an experimental predator exposure and could not be relocated.

Three purple sunstars (mean ±SD diameter: 19.5 ± 1.5 cm) were collected from Broad Cove, St. Philip’s (near Bread and Cheese Cove), on 29 September 2015, and used as predators. They were transported in 75-L bins filled with seawater to the Ocean Sciences Centre (OSC) of Memorial University of Newfoundland, where they were transferred to 30-L glass holding tanks supplied with a continuous flow of seawater pumped in from the adjacent Logy Bay. Sunstars were not fed during the study (29 September to 27 November 2015) to standardize their hunger levels and associated behavioral responses upon contacting sea cucumbers. Sea stars are generally tolerant to food deprivation, with no perceptible physiological or behavioral impacts over periods of at least 2 months (Hancock 1958; Jangoux and van Impe 1977; Rochette et al. 1994; St-Pierre and Gagnon 2015a).

Subjects in the laboratory mesocosm experiment were 40 orange-footed sea cucumbers collected from across the study site, including 15 individuals tested in the field experiment. During the experiment, 2 subjects became moribund and were discarded. Thus, the final sample size for the mesocosm experiment was 38 individuals (mean ±SD contracted body length: 14.6 ± 1.9 cm; range: 10.2–18.6 cm). Sea cucumbers were collected and transported to the lab on 2 and 31 October 2015. During collection, divers detached the sea cucumbers from the seabed by gently rocking them from side to side until all podia disengaged. They were placed in large (75 L), labeled containers filled with seawater, and transported to the OSC. Subjects arrived at the facility within 5 h of collection and were transferred to 30-L glass holding tanks (maximum of 3 individuals per tank) supplied with a continuous flow of seawater from Logy Bay. Sea cucumbers were maintained in holding tanks for 3 days, then moved into the mesocosm (oscillatory wave tank, described below).

### Field experiment

**Figure 1.**
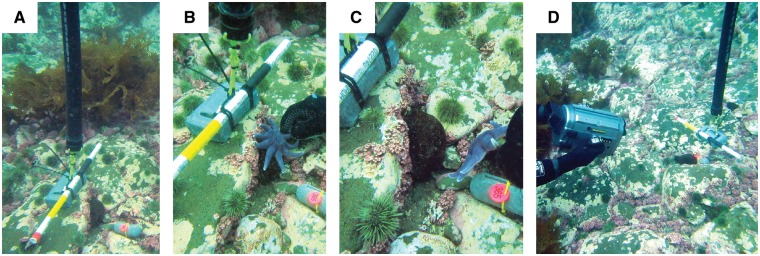
Procedure for experimental tests in the field. (**A**) An URSKI with weight and scale bar was placed close to a tagged orange-footed sea cucumber (*Cucumaria frondosa*). (**B**) After 40 s of baseline video, a purple sunstar (*Solaster endeca*) selected haphazardly from a mesh bag containing 3 sunstars was placed gently on top of the sea cucumber. The sunstar stayed on the sea cucumber for 60 s, untouched by divers. (**C**) The sunstar was removed. (**D**) 120 s of post-predator video was recorded. Either the dive weight with orange tag attached or distinctive patches of red coralline algae carpeting the seabed (such as the patch to which the sea cucumber is attached in panel (C) were used as static reference points to correct body shape variability measurements (see the “Materials and Methods” section for details).

Two trained scientific divers (first and third authors of present article) tested each subject on 9, 17, and 24 October 2015. Each 220-s test was divided into 3 periods: 40-s baseline, 60-s predator exposure, and 120-s post-predator observation (Figure 1). At the beginning of the predator exposure period, 1 experimenter haphazardly selected 1 of the 3 purple sunstars from a mesh bag, placed it on the exposed upper surface of the sea cucumber, and gently held it in place until it attached to the sea cucumber with its tube feet. The 60-s exposure began the moment the sunstar and sea cucumber made contact. The experimenter’s hand never made direct contact with the sea cucumber, and the sunstar was removed after 60 s. The subject’s behavior was video-recorded throughout the test with a submersible, high-definition digital video camera system (Sony HVR-V1 with an Amphibico Endeavor housing; Figure 1D). For each of 6 individuals, the baseline period of 1 of their 3 tests was shorter than 40 s (15–35 s) because of a technical issue with the camera. Once a test was complete, divers moved to the next subject and repeated this process until all subjects had been tested. Subjects were tested in the same haphazard order (shortest itinerary for the divers) on each of their 3 test days. However, because of limited dive time, we tested one end of the site in the morning and the other end in the afternoon and alternated the order from one test day to the next.

We note 2 important points about the predator exposures in this study. First, each sea cucumber’s physical contact with a purple sunstar was relatively spontaneous and brief because of time constraints imposed by scuba diving. However, sudden, short-lived attacks are not unusual; So (2009) reports a tendency in purple sunstars to approach orange-footed sea cucumbers from downstream, likely to facilitate surprise attacks, as seen in other holothurian predators (Kropp 1982). Second, we did not include a positive control in which sea cucumbers were exposed to an innocuous item, instead of a predator, because other research has shown that sea cucumbers respond more strongly to predators than to innocuous items. Legault and Himmelman (1993) found that only 20% of orange-footed sea cucumbers responded to direct contact with a synthetic rug, with a mean (95% CI) reaction time of 223 (97) s. In contrast, all sea cucumbers responded to contact with a purple sunstar, with a mean reaction time of 59 (11) s. The latter study therefore established that the sunstar elicits a significantly stronger antipredator behavior than a positive control.

Hydrodynamic forces were measured continuously with modified underwater relative swell kinetics instruments (URSKIs; Figurski et al. 2011; Figure 1A). An URSKI consists of a submersible accelerometer (Onset HOBO UA-004-64 Pendant G Data Logger) housed in a perforated, cylindrical, 8-cm-long container epoxied to one end of a sealed, slightly positively buoyant, 90-cm-long ABS pipe (8 cm in diameter). The other end of the pipe was tethered with an 18-cm-long twine to eyebolts drilled into the seabed at depths of 5 and 11 m, which approximated the upper and lower extremes of the depth spectrum. In still water, the instrument stood vertically in the water column with the accelerometer at the upper, untethered end, approximately 1.15 m above the seabed. In the presence of water flow, the free end of the instrument, and hence the accelerometer, tilted at a speed, direction, and angle consistent with prevailing flows. The accelerometer recorded its own instantaneous acceleration in the *x*- (vertical), *y*- (horizontal), and *z*- (horizontal) directions every 30 s. The *y*- and *z*-direction data were used to calculate, by trigonometry, instantaneous acceleration vectors indicative of the horizontal (parallel to seabed) flow acceleration to which sea cucumbers were exposed. From this data, we calculated mean hourly flow accelerations at each end of the depth spectrum between 25 September and 31 October 2015 (entire survey duration).

We also measured local hydrodynamic forces by each subject during its 3 tests by placing a portable URSKI on the seabed approximately 30 cm from the subject. This URSKI recorded its own instantaneous acceleration every second throughout the 220-s tests. Instantaneous acceleration vectors were calculated as explained above. Because accelerometers were approximately 1 m above the seabed, hydrodynamic forces measured may have differed, albeit slightly, from those experienced by the sea cucumber 1 m below (Denny 1988; Denny and Wethey 2001). Any difference in flow regimes was deemed inconsequential because: (1) the present study was concerned with effects of relative (not absolute) hydrodynamic forces among test subjects; and (2) all test subjects were laying on the bedrock seabed and hence occupied microhabitats that would have influenced local flows in similar ways.

Activity level in sea cucumbers, like in many other ectothermic marine invertebrates, varies with water temperature (Angilletta et al. 2010; Frey and Gagnon 2015; Kühnhold et al. 2017; Tagliarolo et al. 2018). To ensure that temperature did not confound our analyses, we recorded water temperature at 15-min intervals from 25 September to 31 October 2015 with a temperature logger (Onset HOBO UA-002-64 Pendant; accuracy ±0.5°C) bolted to the seabed at both ends of the depth gradient. Water temperature was similar at both depths throughout the experiment (5 m depth: mean = 9.5°C, SD = 0.7°C, range = 4.6°C; 11 m depth: mean = 9.3°C, SD = 0.7°C, range = 5.0°C). Accordingly, any effect of temperature on antipredator responses was unlikely to differ along the depth gradient studied. The depth of each subject was measured with a handheld gauge (Tusa SCA-360; accuracy ±0.3 m).

### Laboratory mesocosm experiment

**Figure 2.**
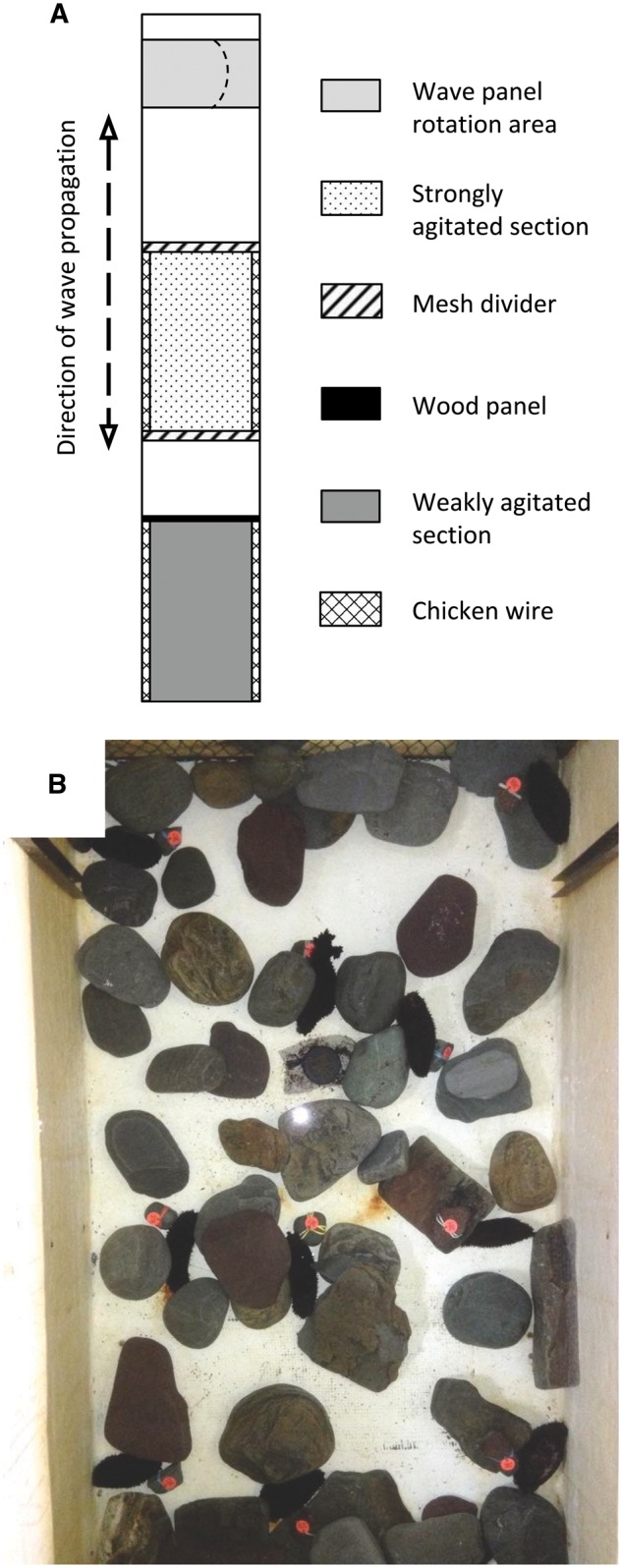
Setup for the mesocosm experiment. (**A**) Top-view schematic of the wave tank depicting the 2 sections, their dividers, and other components of the tank setup (see the “Materials and Methods” section for details). (**B**) Sample video frame from the strongly agitated section. Darkly colored sea cucumbers are visible against the light tank. Small, numbered orange tags were attached to rocks and positioned directly next to each sea cucumber to keep track of their identities.

The laboratory experiment was carried out in an oscillatory wave tank [l × w: 6 × 1 m; see tank details in Frey and Gagnon (2015), St-Pierre and Gagnon (2015b); Figure 2] that mimicked the back-and-forth flow caused by waves in subtidal habitats. One end of the tank contained a rotating panel that generated 15 wave cycles min^−^^1^ at a peak longitudinal velocity of 0.2 m s^−^^1^, as measured with a Doppler current meter (Vector Current Meter; Nortek) held approximately 5 cm above the center of the experimental area (without the structures used to create the microhabitat; see below). These conditions approximated the frequency of waves and average water flow speeds at our study site under moderate wind conditions (Frey and Gagnon 2015; St-Pierre and Gagnon 2015b). Waves propagated into a 1.5-m-long section in the middle of the tank that was demarcated by nylon netting and used as the “strongly agitated” section. Another 1.5-m-long section, located at the end of the tank opposite the wave generating mechanism, was used as the “weakly agitated” section. It was separated from the other sections by a transverse plywood partition that prevented waves from propagating through. To simulate natural seabed heterogeneity in the wave tank, rocks were placed (diameter: 7–33 cm; density: 27.3 rocks m^−^^2^) on the bottom of both sections. Chicken wire was attached to the sides of the tank to prevent sea cucumbers from climbing up the walls. The wave tank was operated as a closed system to hold water level and flow pattern constant. To help maintain a temperature close to that of the sea cucumber’s natural habitat, the tank was drained daily from a depth of 37 cm to a depth of 20 cm (a level that ensured that all sea cucumbers remained submerged) and refilled immediately with seawater pumped in from Logy Bay. Water temperature in the tank was recorded hourly throughout the experiment with a temperature logger (accuracy: ±0.5°C; HOBO Pendant; Onset Computer Corporation). The daily variance in water temperature throughout the experiment (15 October to 27 November 2015) was 0.84 ± 0.92°C (mean ±SD).

The tank was illuminated by indirect, natural light through a window, and by supplemental artificial lighting. There were 2 sources of artificial lighting: (1) 2 120-cm-long, 32-W Sylvania Octron fluorescent tubes (4100 K, FO32/841/ECO) and 1 120-cm-long, 34-W General Electric Cool White (4100 K, Hg, F34 CW WM ECO EX) fluorescent tube suspended from the ceiling approximately 2 m above the wave tank; and (2) 2 500-W Globe Electric Company Inc. model 04787 and 2 150-W Sylvania model 4406156R halogen bulbs (1300 W in total) on a short (1.75 m) stand placed outside of the wave tank approximately 1.5 m from the 2 working sections. Fluorescent tubes were permanently turned on, as per building code, whereas halogen bulbs were turned on only during video recording of subjects to provide adequate light for crisp video footage. Illuminance from the fluorescent tubes at the water surface, based on 6 measurements with a portable lux meter (accuracy: ±10 lx; Hi97500; Hanna Instruments), was 216.6 ± 18.6 lx (mean ±SD).

Before testing began, we stopped waves and moved subjects into the tank section that simulated their site of origin in the field: those from the deeper (>8 m) end of the gradient went into the weakly agitated section of the tank, whereas those from the shallower (<8 m) end went into the strongly agitated section. Each section could accommodate up to 10 sea cucumbers at a time. This density was similar to the highest densities observed at our field site; prior to testing, up to 6 additional sea cucumbers had been removed from within a 1-m radius of each subject in the field experiment. We gave subjects 60–100 min to attach to the substratum before gradually raising the flow to 15 waves min^−^^1^. Subjects were individually marked by placing unique numbered lead weights beside them, and by repositioning weights throughout the experiment whenever subjects moved (movement was infrequent). Before testing began, all subjects were acclimated to the wave tank for 3 days. During acclimation to the strongly agitated section, 8 of the 15 individuals that were previously tested in the field experiment moved around the tank when experimenters were not present. Consequently, we could not keep track of their identities and were left with an insufficient sample from which to draw behavioral comparisons between the lab and field.

Pilot testing was carried out on 10 sea cucumbers that were not tested in the mesocosm experiment to confirm previously reported reactions to a positive control (Legault and Himmelman 1993); each was observed for 30 min following 30 s of contact with an empty neoprene diving glove. The sea cucumbers contracted slightly following stimulation, but did not alternate between elongation and contraction, as seen in antipredator responses (Francour 1997), and none of them detached. In the mesocosm, we tested each experimental subject’s response to a predatory sunstar 6 times, at 3- or 4-day intervals. After testing each subject 3 times, half of the subjects from each section of the wave tank were selected using a random number generator (Haahr 2018) and transplanted to the opposite section for their final 3 tests. Those that were not transplanted received a sham disturbance, where they were lifted out of the water and set back down in a different location within the same section. The test procedure was similar to that in the field experiment, except that the duration of each test was not limited by scuba diving constraints. Therefore, each test included a 180-s baseline, 60-s predator exposure, and 60-min post-predator observation period. Predator exposure was the same as in the field experiment, except that we used the same sunstar (selected haphazardly) to test all subjects in each section of the wave tank on a given test day. Once the predator was removed from a subject, we began the baseline period of the next subject. Therefore, the 60-min post-predator observation periods in each section of the tank overlapped.

Subjects from a given section of the wave tank were tested in a haphazard order during their first test, and then in the same order for tests 2 and 3. Following the transplant, subjects from a given section were tested in a new haphazard order for the fourth test, and then in the same order for tests 5 and 6. The 2 sections were always tested on the same day, but the order of testing alternated from one test day to the next. Tests were video-recorded with the same camera used in the field experiment (without its amphibious housing). The camera was mounted 1.3 m above the wave tank and pointed downward such that its field of view captured the entire focal section of the tank.

### Analyses

In the field experiment, we defined antipredator response as an increase in body shape variability in response to physical contact with a sunstar. Using image analysis software (Tracker, Douglas Brown, version 4.91), we measured the subject’s length and width (1-pixel accuracy) at 5-s intervals throughout the baseline and post-predator observation periods. Length was the longest distance between the subject’s anterior and posterior ends, while width was the length of the line perpendicular to the body length vector that maximized the distance between the subject’s 2 sides. We calculated the ratio between length and width at each 5-s interval, as well as the standard deviation of the resulting length-to-width ratios for both the baseline and post-predator observation periods. We refer to the standard deviation of the length-to-width ratios as “body shape variability.” Only 1 subject detached from the seabed during testing, so we did not analyze this aspect of the antipredator response in the field experiment.

The video camera moved slightly with wave action throughout the field experiment. This inevitable motion may have changed the camera’s perspective of the subject and influenced measures of length and width from one 5-s interval to the next. We therefore applied a correction procedure. At each 5-s interval, we measured the length and width of a static object located immediately beside the subject, which had comparable size and orientation. Such objects were either the lead weight used to identify the subject or, if the weight was not entirely within the field of view, a rock with distinctive rhodolith markings (as seen in Figure 1C). Changes in length or width of the object caused by any change in camera angle, relative to corresponding measurements obtained from the first 5-s interval of the test, were used to calculate correction factors for length and width during that time interval.

We used linear regression to test whether a subject’s depth at the field site influenced the strength and variability of the hydrodynamic forces surrounding it. We defined strength as the median of the 660 accelerations recorded during a subject’s 3 tests, and variability as the interquartile range. We used non-parametric measures of strength and variability because histograms showed that the acceleration data were positively skewed.

Before examining antipredator responses, we tested for a relationship between the sea cucumbers’ natural activity levels and their local hydrodynamic environment and time of day. On a given test day, we defined natural activity level as each subject’s body shape variability during the baseline observation period. We used a linear mixed effects model (LMM) to test whether a subject’s baseline body shape variability covaried with the strength of hydrodynamic forces during its 220-s test period and the time of day (hours since midnight), with subject identity included as a random intercept to account for multiple measures from the same individual. This test showed that flow strength, but not time of day, was a significant predictor of baseline body shape variability (LMM: effect of time: *t*_39_* *=0.908, *P *=* *0.369; effect of flow strength: *t*_39_ =2.21, *P *=* *0.0329). Because natural activity levels varied with the strength of hydrodynamic forces, we next determined whether a subject’s baseline body shape variability correlated with its body shape variability during the post-predator observation period. A Pearson correlation showed that body shape variability during the post-predator period was not related to body shape variability during the baseline period (*t*_13_ =1.26, *P *=* *0.229). Thus, we did not include baseline body shape variability in analyses of behavior during the post-predator observation period.

Subsequently, we tested whether subjects increased their body shape variability in response to the predatory sunstar. This was done by calculating each subject’s average body shape variability among its 3 baseline periods and, separately, among its 3 post-predator observation periods, and then comparing the average baseline and post-predator observation periods among subjects using a paired *t*-test. To test the hypothesis that antipredator behavior is related to the strength of local hydrodynamic forces, we used linear regression to compare a subject’s average body shape variability in its 3 post-predator observation periods to the median strength of the hydrodynamic forces recorded during those periods. Tests were 2-tailed, and results were considered statistically significant when *P *≤* *0.05.

In the laboratory mesocosm experiment, we defined an antipredator response as either an increase in body shape variability or detachment from the substratum in response to contact with a sunstar. We calculated each subject’s body shape variability during the baseline and post-predator observation periods following the same methods as described for the field experiment. However, we measured each subject’s length and width at 10-s intervals (instead of 5-s intervals) and we did not apply a correction procedure to the measurements since the camera did not move during tests. We noted whether subjects detached from the substratum during the test periods. One individual, which was in the weakly agitated section of the tank throughout the experiment, climbed the side of the tank and was out of the video frame during all 3 tests of the post-transplant period. Data from this subject were not included in analyses involving the post-transplant period.

The mesocosm was operated as a closed system, so it is possible that chemicals released by the sunstar or sea cucumbers accumulated in the tank and affected antipredator responses. Such accumulation could potentially create test order effects that could make the responses of different subjects non-independent. Before proceeding with analyses, we tested whether the order in which a subject was exposed to the sunstar on a given day (i.e., 1–10) affected the strength of its antipredator responses. The order of testing did not affect a subject’s mean body shape variability during its 3 tests from before the reciprocal transplant (linear regression: *t*_34_ =0.086, *P *=* *0.932) or during its 3 tests after the reciprocal transplant (linear regression: *t*_34_ =1.00, *P *=* *0.324). It also did not affect the number of tests in which an individual detached during its 3 pre-transplant tests (linear regression: *t*_36_ =0.57, *P *=* *0.574) or its 3 post-transplant tests (linear regression: *t*_36_ =0.70, *P *=* *0.488).

After establishing that behavior was independent of presentation order, our first question was whether body shape variability changed in response to predator exposure. We performed a paired *t*-test, just as for the field experiment, but calculated each individual’s average body shape variability across all 6 baseline and post-predator observation periods. A few subjects climbed the walls of the tank and partially left the video frame, thus preventing body shape variability from being measured (31 baseline measures from 14 subjects and 33 post-predator measures from 17 subjects). These tests were excluded from the calculation of average body shape variability. However, this usually occurred in only some of a subject’s 6 tests, so it was still possible to calculate an average for that subject from its remaining tests. To evaluate whether subjects had a higher probability of detaching from the substratum in response to the sunstar, we used a Wilcoxon signed ranks test to compare the number of baseline periods (0−6) and the number of post-predator observation periods (0−6) in which each subject detached.

Our next question was whether antipredator responses differed between the 2 hydrodynamic environments. Dependent variables were the average body shape variability and total number of tests in which a subject detached. The independent variable was “hydrodynamic condition” (weakly agitated or strongly agitated). Each variable was tested separately during the pre-transplant and post-transplant periods because half of the individuals changed hydrodynamic conditions during the transplant. Therefore, the number of tests in which a subject detached varied from 0 to 3 in both pre-transplant and post-transplant analyses. Body shape variability was compared between hydrodynamic conditions with unpaired *t*-tests, and the number of detachments was compared with Mann–Whitney tests. Because we used 4 independent tests to address the same question, we controlled for inflated risk of type I error using the sequential Bonferroni method (Holm 1979). We present only the adjusted *P*-values.

Our last question was whether sea cucumbers alter antipredator behavior in response to a sudden change in hydrodynamic forces. Sea cucumbers experienced 3 types of hydrodynamic shifts during the transplant experiment: strongly to weakly agitated water, weakly to strongly agitated water, and no change. Splitting the no change group into the 2 types of sham disturbance (strongly to strongly agitated and weakly to weakly agitated) would add no additional information about environmental change but would cost statistical power due to our small sample size. The dependent variable was the change in the total number of tests in which a subject detached (i.e., post-transplant – pre-transplant), and thus ranged from −3 to 3. The change in detachment frequency was compared among the 3 transplant conditions using a 1-way ANOVA with post hoc pairwise comparisons. We corrected for multiple post hoc comparisons (Holm 1979) and presented only the adjusted *P*-values. We did not analyze body shape variability for this question because there was no effect of hydrodynamic condition on body shape variability in the previous set of analyses (see the “Results” section). Effect sizes for all parametric tests were measured using Cohen’s *d* (Cohen 1988; Sawilowsky 2009). All analyses were conducted in R (version 3.3.1; R Core Team 2016).

### Ethical note

All research practices complied with the “Guidelines for the treatment of animals in behavioral research and teaching” set forth by the Animal Behavior Society (Buchanan et al. 2012), and with the “Guide to the care and use of experimental animals” set forth by the Canadian Council on Animal Care (Olfert et al. 1993). These experiments were conducted under Memorial University of Newfoundland animal care and use protocol number IACC 15-05-PG.

## Results

### Field experiment

**Figure 3.**
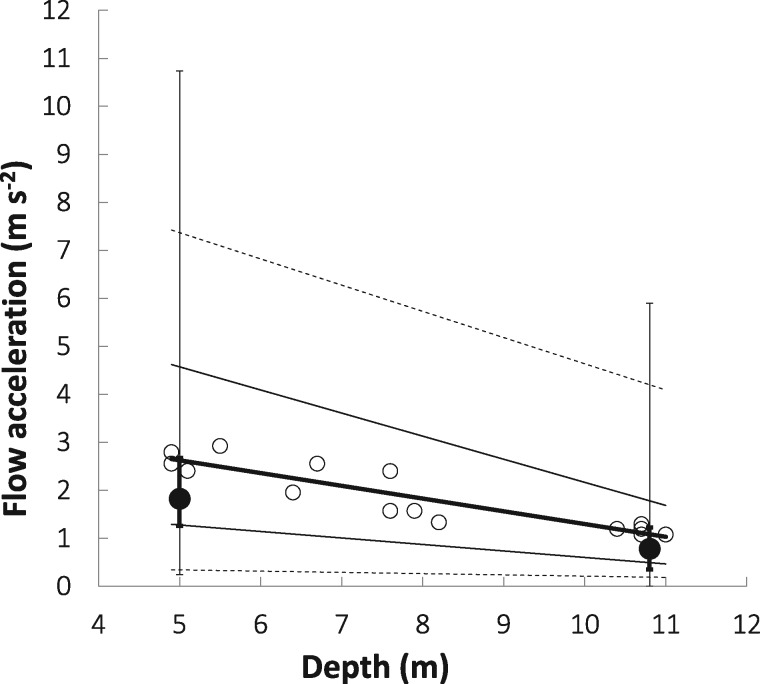
Water flow acceleration by depth for 15 orange-footed sea cucumbers. Each open circle shows the median instantaneous acceleration at a given subject’s location and the thick line shows the line of best fit among these points. The 2 thin solid lines are the lines of best fit for the 10th (lower line) and 90th (upper line) percentile values of the acceleration values calculated at each of the 15 subjects’ locations, and the outermost dashed lines are the lines of best fit for the minimum (lower line) and maximum values at each of these locations. The closed circles are the median of the hourly mean flow accelerations at the 2 ends of the study site from the long-term data, and the associated error bars show the inter-quartile ranges (thick error bars) and ranges (thin error bars) for the long-term acceleration measurements.

Sea cucumbers were distributed along a pronounced hydrodynamic gradient that covaried with depth (Figure 3). Shallower individuals experienced stronger (linear regression: *F*_1,__13_ =59.2, *P *<* *0.001, *R*^2^ =0.82) and more variable (*F*_1,__13_ =66.9, *P *<* *0.001, *R*^2^ =0.84) flow accelerations than deeper individuals. Long-term flow acceleration data were consistent with this pattern; stronger and more variable hourly accelerations occurred at the shallow extreme, whereas weaker, less variable flow accelerations occurred at the deep extreme. Interestingly, the range of flow accelerations observed during short tests spanned most of the range of the long-term accelerations observed throughout the entire study (Figure 3).

Exposure to sunstars increased the body shape variability of the 15 orange-footed sea cucumbers. Body shape variability was significantly greater during the post-predator observation periods (mean ±SD: 0.20 ± 0.04) than during the corresponding baseline periods (mean ±SD: 0.15 ± 0.07; paired samples *t*-test: *t*_14_ =2.6, *P *=* *0.021, *d *=* *0.7). Antipredator behavior was not related to the strength of the subject’s local hydrodynamic forces, since average body shape variability during the subject’s 3 post-predator observation periods (one from each testing day) did not covary with the median instantaneous flow acceleration recorded throughout the 3 tests (linear regression: *F*_1,__13_ =0.0, *P *=* *0.902, *R*^2^ <0.01).

### Laboratory mesocosm experiment

Exposure to a sunstar elicited antipredator behavior among the 38 orange-footed sea cucumbers tested. Specifically, the average body shape variability for a subject’s 6 post-predator observation periods (mean ±SD: 0.39 ± 0.18) was significantly greater than the average body shape variability observed for its 6 baseline periods (mean ±SD: 0.09 ± 0.05; paired samples *t*-test: *t*_37_ =10.7, *P *<* *0.001, *d *=* *1.7). Furthermore, no sea cucumbers detached during any of their baseline periods, whereas 30 of the 38 sea cucumbers detached during at least 1 of their 6 post-predator observation periods (median [IQR] number of post-predator observation periods in which a sea cucumber detached: 1.5 [1–4] tests; Wilcoxon signed ranks test: *V *=* *465, *P *<* *0.001).

Body shape variability did not differ between the weakly agitated (mean ±SD: 0.41 ± 0.24) and strongly agitated (mean ±SD: 0.43 ± 0.17) conditions during the pre-transplant period (unpaired t-test: *t*_34_ =0.29, *P*_adj_* =*>0.999, *d *=* *0.10), or between the weakly agitated (mean ±SD: 0.43 ± 0.22) and strongly agitated (mean ±SD: 0.33 ± 0.22) conditions during the post-transplant period (*t*_34_ =1.4, *P*_adj_* *=0.526, *d *=* *0.46). Similarly, the number of tests in which a sea cucumber detached did not differ between the weakly agitated (median [IQR]: 1 [0–1.5] tests) and strongly agitated (median [IQR]: 1 [0.5–1] tests) conditions during the pre-transplant condition (Mann–Whitney test: *W *=* *163.5, *P*_adj_ =>0.999). However, sea cucumbers detached more often in the weakly agitated condition (median [IQR]: 2 [1–3] tests) than in the strongly agitated condition (median [IQR]: 0 [0–1] tests) during the post-transplant period (*W *=* *273, *P*_adj_ =0.020).

**Figure 4.**
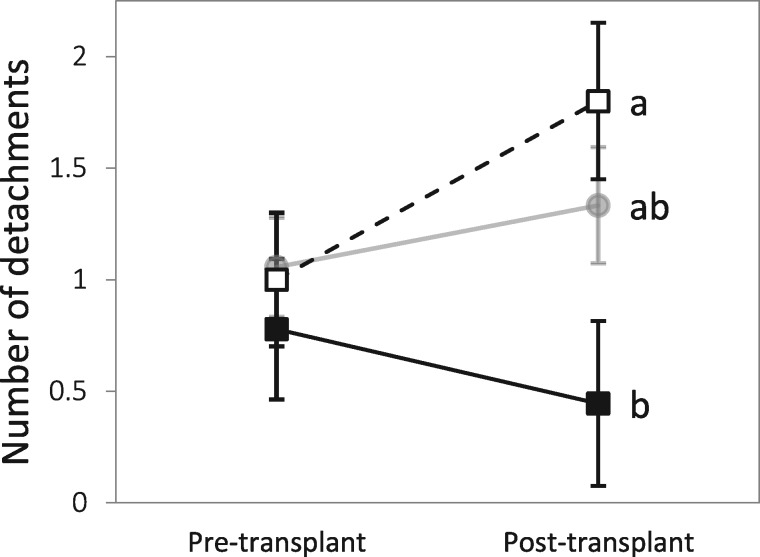
Effect of reciprocal transplants on the number of post-predator observation periods in which a subject detached from the substratum. Open squares connected by a dashed line represent subjects moved from strongly to weakly agitated conditions. Solid squares connected by a solid line represent subjects moved from weakly to strongly agitated conditions. Solid circles connected by a faded gray line represent control subjects moved to another location within the same environment (sham disturbance). Change in behavior (post-transplant – pre-transplant) was compared among the 3 transplant treatments with a 1-way ANOVA. Treatments with different letters are statistically different, as indicated by post-hoc, pairwise comparisons corrected for type I error (Holm 1979).

The type of environmental change experienced during the reciprocal transplant had a significant effect on the change in detachment frequency (1-way ANOVA: *F*_2,__35_ =3.9, *P *=* *0.030, $R_{\text{adj}}^{2}$ =0.14). Specifically, individuals that had been moved from strongly to weakly agitated water showed a significant increase in detachment frequency relative to individuals that moved from weakly to strongly agitated water (post hoc comparison: *P*_adj_* *=0.030; Figure 4).

## Discussion

Contrary to our first hypothesis, the strength of antipredator responses did not differ between orange-footed sea cucumbers in different hydrodynamic environments. However, sea cucumbers became less likely to detach from the substratum when hydrodynamic forces increased, and more likely to detach when hydrodynamic forces decreased, as per our second hypothesis. These findings confirm that sea cucumbers alter their threshold for detaching when environmental conditions change. While many studies report behavioral and morphological plasticity in vertebrates (e.g., tree-lizards, *Urosaurus ornatus*, Moore et al. 1998; Trinidadian guppies, *Poecilia* *reticulata*, Kolluru et al. 2007), and morphological plasticity in marine invertebrates (Bourdeau and Johansson 2012), few have demonstrated behavioral plasticity in marine invertebrates (Padilla and Savedo 2013). Exceptions from outside the echinodermata include soft-shell clams (*Mya areneria*), which bury deeper into the sediments when exposed to predators (Whitlow 2010), mussels (*M. edulis*), which cluster together in response to a predator (Côté and Jelnikar 1999), and coral polyps (*Porites compressa*), which withdraw into their protective skeletons for longer when predators are near (Gochfeld 2004). There are also examples of behavioral plasticity in echinoderms. For example, sand dollar *Dendraster excentricus* larvae cease growing and begin cloning upon detecting mucus from predatory fish, as smaller body size affords a temporary refuge from predation (Vaughn and Strathmann 2008). These larvae also avoid predatory fish cues by shifting their distribution in the water column (Arellano et al. 2012). Likewise, sea urchins *Psammechinus miliaris* exhibit variable morphology of feeding structures and foraging behaviors in different trophic environments (Hughes et al. 2012). In these examples, behavioral plasticity is exhibited in response to the animal’s biotic environment. To our knowledge, our study demonstrates, for the first time, plasticity in the antipredator behavior of a slow-moving marine invertebrate that is induced by changes in its abiotic environment. Thus, it joins a growing literature that is uncovering the myriad adaptations that echinoderms and other slow-moving and sessile marine organisms demonstrate in response to environmental change.

The strength of sea cucumbers’ antipredator responses did not differ between the weakly and strongly agitated conditions of the mesocosm experiment before transplants, or in relation to the hydrodynamic forces in the field experiment. It is possible that sea cucumbers in the field had insufficient time to produce a complete antipredator response. Indeed, changes in body shape variability between the baseline and post-predator observation periods were more than 6 times greater in the mesocosm experiment, where the post-predator observation period lasted for 60 min, than in the field experiment, where it lasted for only 2 min. As we and others have observed (Legault and Himmelman 1993), a sea cucumber’s antipredator response can last for more than an hour after the sunstar is removed. Although the field experiment enabled us to observe antipredator behavior in the wild, and to characterize the hydrodynamic conditions experienced by sea cucumbers, the short sampling periods imposed by scuba diving may have obscured a more subtle relationship between the strength of a sea cucumber’s antipredator behavior and local hydrodynamic conditions. Also, no wave tank can perfectly simulate hydrodynamic forces generated in the ocean. Hydrodynamic forces in intertidal and shallow marine environments are highly variable spatially and temporally (Denny 1988), which limits reproducibility in wave tanks. While wave and flume tank experiments are commonly used to isolate effects of hydrodynamic forces on benthic organisms (e.g., Harvey et al. 1995; Kawamata 1998; Gagnon et al. 2003; Peralta et al. 2008; Morse and Hunt 2013), test subjects in the present study may have habituated more quickly to the artificial rhythm of hydrodynamic forces in the tank. Since hydrodynamic forces were both stronger and more variable in the shallow environment, it would be valuable to assess the independent and combined effects of flow strength and variability on antipredator responses.

Alternatively, a relationship between antipredator responses and hydrodynamic forces may emerge only in response to recent changes in hydrodynamic conditions and may then weaken over time as the novelty of change disappears. Animals that show behavioral plasticity in response to sudden environmental change often respond strongly at first, but then show diminished responses over time (Tuomainen and Candolin 2011). Marmosets (*Callithrix penicillata*), for example, show increased vigilance in novel environments, but then show diminished vigilance with repeated exposures to that same environment (Barros et al. 2008). Some marine invertebrates also exhibit behavioral plasticity that diminishes with prolonged exposure to new environments. Copepods (*Centropages hamatus*) display heightened escape responses immediately following the onset of increased water turbulence, but rapidly habituate and begin showing the same responses that are seen in non-turbulent water (Hwang et al. 1994). Therefore, differences in the antipredator behavior of orange-footed sea cucumbers may reflect short-term responses to recent environmental change, rather than prolonged responses to stable differences in local hydrodynamic conditions.

Sea cucumbers in the mesocosm experiment responded to changes in hydrodynamic forces, yet the degree to which this response occurs in the wild remains unknown, and further research is required to determine whether such plasticity is adaptive. We suggest that individuals experiencing a shift to less agitated waters express stronger antipredator behavior in response to an attack because the associated risk of being washed away is reduced. While a nearby predator may seem like the greater risk, the chemical cues sea cucumbers use to detect predators indicate only that a predator is nearby, and not necessarily that one is hunting (Legault and Himmelman 1993). Sea cucumbers are often densely aggregated, and hence even if a predator is hunting, an individual sea cucumber’s risk of being targeted during an attack may be quite low (Hamilton 1971; Legault and Himmelman 1993; So 2009). Furthermore, foraging efficiency of predators may be reduced under stronger hydrodynamic forces. Turbulent water flows may dissipate odor plumes, making it more difficult for mobile predators that rely on chemosensory mechanisms to detect prey; predators may also experience higher mortality rates than their prey under relatively strong hydrodynamic forces (Powers and Kittinger 2002; Petes et al. 2008). Marine invertebrates often “scale” antipredator reactions to the level of predation risk (Thomas and Gruffydd 1971; Smee and Weissburg 2006; Selander et al. 2011). A good example is the sea pen, *Ptilosarcus gurneyi*, which becomes more likely to burrow in the sediment when exposed to sea stars that are more specialized predators, and will only burrow upon physical contact, not in response to waterborne predatory cues (Weightman and Arsenault 2002). Increasing hydrodynamic forces might thus provide sea cucumbers with a cue of diminished predation risk, causing a shift toward a lower likelihood of detaching. If detachment increases a sea cucumber’s likelihood of escaping a predatory attack, as previous studies suggest (Kropp 1982; Legault and Himmelman 1993; So 2009), our results may indicate that a trade-off is imposed by detaching and floating in stronger flow environments.

Sea cucumbers in the wild experience changing hydrodynamic conditions, such as those simulated in our experiment, in at least 2 situations. First, newly settled orange-footed sea cucumbers migrate from shallow to deeper waters over a period of several months, beginning in autumn (Hamel and Mercier 1996). Although our transplants occurred over a much shorter period, the mechanisms underlying the phenotypic plasticity observed in our experiment may have evolved in the context of migration. Second, severe storms can dramatically increase water flow within a few hours (Harmelin-Vivien 1994), which approximates the timescale of change simulated in our mesocosm experiment. The rapid change created by storms could also explain why sea cucumbers have evolved the capacity to rapidly alter antipredator responses, since this could mitigate the risk of being washed away into unsuitable environments. Some evidence suggests that when an orange-footed sea cucumber is swept onto soft substrata, where it is unable to attach, it becomes emaciated and eventually dies (So et al. 2010). If so, being swept ashore is not the only risk associated with detachment; sea cucumbers could also be displaced into unsuitable habitats at the deeper end of their distribution. Thus, plasticity in antipredator responses may allow orange-footed sea cucumbers to mitigate the risk of being displaced into unsuitable habitat. Future research should assess the adaptive value of this plasticity by testing whether strong antipredator responses, such as detaching, lead to a higher risk of stranding and mortality on a seabed with strong hydrodynamic forces.
